# Uncovering the Complex Transcriptome Response of *Mytilus chilensis* against Saxitoxin: Implications of Harmful Algal Blooms on Mussel Populations

**DOI:** 10.1371/journal.pone.0165231

**Published:** 2016-10-20

**Authors:** Camille Detree, Gustavo Núñez-Acuña, Steven Roberts, Cristian Gallardo-Escárate

**Affiliations:** 1 Laboratory of Biotechnology and Aquatic Genomics, Interdisciplinary Center for Aquaculture Research (INCAR), University of Concepcion, Concepción, Chile; 2 School of Aquatic and Fishery Sciences (SAFS), University of Washington, Seattle, United States of America; Helmholtz-Zentrum fur Ozeanforschung Kiel, GERMANY

## Abstract

Saxitoxin (STX), a principal phycotoxin contributing to paralytic shellfish poisoning, is largely produced by marine microalgae of the genus *Alexandrium*. This toxin affects a wide range of species, inducing massive deaths in fish and other marine species. However, marine bivalves can resist and accumulate paralytic shellfish poisons. Despite numerous studies on the impact of STX in marine bivalves, knowledge regarding STX recognition at molecular level by benthic species remains scarce. Therefore, the aim of this study was to identify novel genes that interact with STX in the Chilean mussel *Mytilus chilensis*. For this, RNA-seq and RT-qPCR approaches were used to evaluate the transcriptomic response of *M*. *chilensis* to a purified STX as well as *in vivo Alexandrium catenella* exposure. Approximately 800 million reads were assembled, generating 138,883 contigs that were blasted against the UniProt Mollusca database. Pattern Recognition Receptors (PRRs) involved in mussel immunity, such as Toll-like receptors, tumor necrosis factor receptors, and scavenger-like receptors were found to be strongly upregulated at 8 and 16 h post-STX injection. These results suggest an involvement of PRRs in the response to STX, as well as identifying potential, novel STX-interacting receptors in this Chilean mussel. This study is the first transcriptomic overview of the STX-response in the edible species *M*. *chilensis*. However, the most significant contribution of this work is the identification of immune receptors and pathways potentially involved in the recognition and defense against STX’s toxicity and its impact of harmful algae blooms on wild and cultivated mussel populations.

## Introduction

Saxitoxin (STX) is a marine toxin that causes paralytic shellfish poisoning, generating detrimental effects in a wide range of marine species, including in organisms ultimately consumed by humans [1]. This toxin is a tetrahydropyran comprised of 26 structural analogues [2]. STX production is mainly related to dinoflagellates of the *Alexandrium* and *Gymnodinnium* genera, which are often associated with harmful algal bloom episodes [1, 3]. These blooms have become more frequent in recent decades, increasing the instances of paralytic shellfish poisoning in natural populations [4].

Among paralytic shellfish poisoning cases derived from harmful algal blooms, STX, which is lethal due to a carbamate-mediated mechanism, is the most common causative toxin [5]. Specifically, STX is a neurotoxin that blocks sodium conduction in nerve and muscular fibers by targeting voltage-dependent sodium channels [6]. Furthermore this toxin can also block voltage-dependent calcium and potassium channels [7].

STX affects many marine species through dinoflagellate ingestion, particularly for suspension-feeding organisms such as bivalves [8]. Physiologically, STX may induce declined reproduction and growth rates, especially in marine bivalves, and could be a major cause of mortality in natural populations [9, 10]. The sublethal physiological responses of marine species to toxins such as STX include reduced filtration rates and changes in feeding and respiration [11, 12]. Additional effects in bivalves can include valve closure, adductor muscle paralysis, mantle retraction, mucus production, and variations in cardiac activity [8, 13].

In turn, teleost fish suffer genotoxicity, cytotoxicity, and oxidative stress after STX exposure [14, 15]. Notably, the genotoxicity induced by STX or harmful algal blooms is not only related to fish. Mat et al. (2013) demonstrated that exposure of the Pacific oyster *Crassostrea gigas* to *Alexandrium* genera microalgae, which contain paralytic shellfish toxins, induces genotoxicity as well as mitochondrial transcriptional repression and the activation of immune response machinery [16]. Recent *in vitro* and *in vivo* studies in the muscle of *Mytilus chilensis* showed that STX exposure induces the upregulation of 13 candidate genes involved in oxidative stress and innate immunity, including Toll-like receptors (TLRs) and C-type lectins [17, 18]. Nonetheless, the pathway and transmembrane receptors that recognize and respond to STX and/or *A*. *catenella* exposure are still unclear in marine species.

The innate immune response, the first barrier of defense against pathogens, includes a diverse group of genes (receptors) referred to as pattern recognition receptors (PRRs). These receptors are transmembrane, intra- or extracellular, and interact with pathogen associated molecular patterns (PAMPs). The most studied pathway in this process is composed of TLRs [19–21], and is generally connected to the apoptosis pathway, regulating part of the homeostasis mechanism and programmed cell death [22]. The innate immune system of mussel species has been studied utilizing diverse approaches, including microarray runs [23] and pyrosequencing against pathogens [24].

Nevertheless, there are no studies that identify and characterize the involvement of all molecular receptors that may be involved in the recognition of a paralytic shellfish poison such as STX. Therefore, the aim of this study was to evaluate the impacts of the purified STX and whole *A*. *catenella* on the transcriptome of *M*. *chilensis* to determine the receptors and pathways related to STX responses. The unpredictable red tide in Chile’s coastal waters, which has recently killed tons of clams and mussels, requires increasing research efforts to understand the molecular mechanisms underlying and their impacts of harmful algae blooms on wild and cultivated mussel populations.

## Materials and Methods

### Sample collection for transcriptomic sequencing

**Fig 1:**
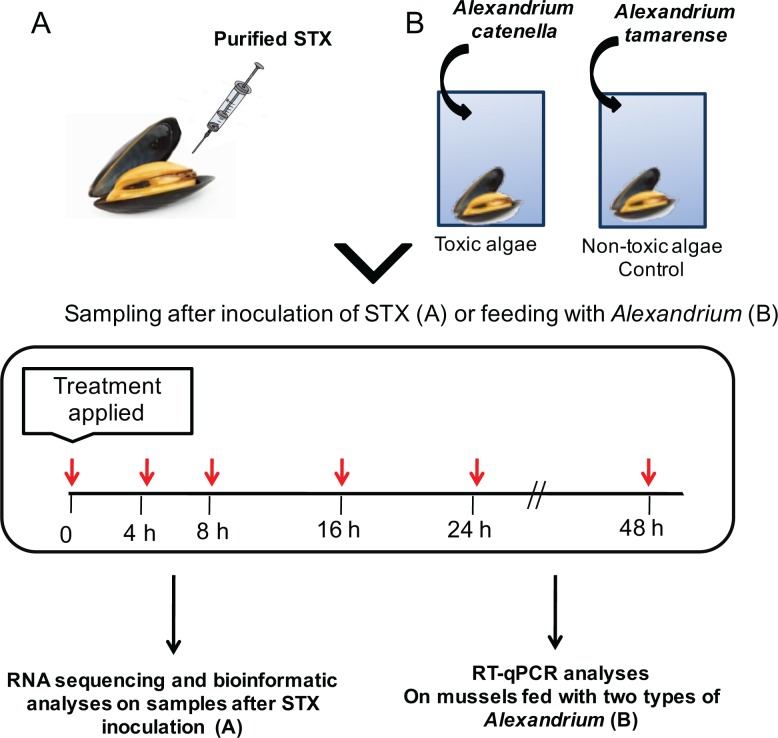
Schematic workflow used in the present study. The experimental design was composed by two separated experiments. (A) An evaluation of the whole transcriptome was performed after direct injection of purified Saxitoxin in mussels, followed by RNA sequencing using the HiSeq2000 platform (Illumina, San Diego, USA) and bioinformatics analysis with the commercial CLC Genomic Workbench software (CLC Bio, Denmark). Complementary to this, (B) a validation assay was performed by exposing mussels to toxic *Alexandrium catanella* and *Alexandrium tamarense*, followed by evaluating the expression levels of selected transcripts through RT-qPCR.

All experiments were conducted at the University de Concepcion (Chile) in accordance with regulations set out by the University. Forty mussels were collected in the Caleta Coliumo, Tomé, Bio Bio Region, Chile (36°32'S—72°57'W) and maintained at 14°C in seawater with constant aeration. No permits were required to collect *Mytilus Chilensis* and the site study does not involve endangered or protected species. After one week of acclimatization, all mussels were weighed and then injected with a purified stock of 80 μg STX / 100 g of wet meat (maximum concentration that can be safely consumed by humans). This stock was injected into the abductor muscle of animals using sterile syringes and PBS as a solvent. Six time points were established to evaluate the response of mussels to STX: 0 h (samples collected immediately after injection), 4, 8, 16, 24, and 48 h post-injection, unfortunately no continuous control was performed during this experiment. At each time point, five mussels were collected (Fig 1). Approximately 1 mL of hemolymph was collected from all sampled individuals using sterile syringes. Hemocytes were obtained through centrifugation at 1200 x *g* for 20 min at 4°C.

### Total RNA extraction and transcriptome sequencing

Total RNA from hemocyte samples was obtained using the RiboPure kit (Ambion, Life Technologies, USA) following the manufacturer’s instructions. Quantity, purity, and quality of isolated RNA were measured in the TapeStation 2200 (Agilent Technologies Inc., USA) using the R6K reagent kit according to the manufacturer’s instructions. Total RNA was pooled from all individuals at the same time point for further analyses. Two different pools were sequenced at each time point. Subsequently, double-stranded cDNA libraries were constructed using the TruSeq RNA Sample Preparation kit v.2 (Illumina, USA) and sequenced with the HiSeq 2000 platform (Illumina, USA) using 2 x 101 paired-end read sequencing runs. The raw sequences were deposited in the Sequence Read Archive (SRA) (http://www.ncbi.nlm.nih.gov/sra) under the accession number SRR3591491.

### Bioinformatics analyses and *in silico* gene expression analyses

Bioinformatics analyses were carried out using the CLC Genomics Workbench v.9 software (CLCBio, Denmark). *De novo* assembly was performed from the transcriptome of six *M*. *chilensis* pools (N = 5 mussels). The assembly parameters used were as follows: mismatch cost = 2, deletion cost = 3, insert cost = 3, minimum contig length = 200 bp, and trimming quality score = 0.05. Transcriptome annotation was based on a BLASTx analysis against the UniProt Mollusca database. Transcripts were annotated according to Gene Ontology terms using the Blast2GO v.3 software (Biobam, Spain). The REVIGO webpage was used according to the developer’s instructions to evaluate the functional activities of the annotated transcripts and to perform enrichment analyses of Gene Ontology terms [25]. REVIGO charts were constructed for biological processes, molecular functions, and cellular components.

*In silico* gene expression analyses were conducted for each library from each time point using the CLC Genomic Workbench software (CLCBio, Denmark). The RNA sequencing (RNA-seq) module was used to calculate the transcripts per kilobase million (TPM) values of each library. Then, values were normalized by scaling, and proportional statistical analyses were conducted to calculate the fold-changes of each transcript as compared to the control using the same software. The scaling normalization method consisted in multiplying the expression values by a constant (the mean expression in this case) and value used as a reference (value after the normalization) was the median mean. The statistical analyses comprise a Kal’s test, and p-value correction based on False Discovery rate (FDR). Transcripts with fold changes > |4| and FDR-corrected p-values lower than 0,05 were used for further analyses. Hierarchical clustering of TPM values was conducted to construct heatmaps, visualize expressional differences, and to select contig clusters among gene expression data according to Manhattan distances.

To identify putative gene pathways related to the contigs of obtained clusters, the KEGG Automatic Annotation Service (KAAS) was used, and the most representative pathways were identified for each cluster. This annotation was conducted using contigs of each cluster as query sequences, and the whole KEGG database as a reference. Most represented KEGG pathways by cluster were obtained by counting the number of hits by GO term, which were grouped according to the pathways that are involved.

On the other hand, a specific analysis was conducted in two selected pathways according to the interest for this study: the TLR and apoptosis pathways. For this, the KEGG pathways database was used to download all gene sequences related to both pathways. Then, sequences from the annotated contigs that were related to both pathways were extracted and kept separately in two independent datasets. RNA-seq analyses, statistical analysis, and hierarchical clustering were performed using the same methods previously described for the complete library datasets.

Additionally, contigs annotated as “receptors” and “channels” in the blast-hit list were extracted, and expression levels were calculated and visualized using the same methodology as above.

### Experimental design for RT-qPCR gene transcription analyses

Two hundred *M*. *chilensis* individuals were separated into six groups for microalgae feeding assessments. Three challenge groups were fed with *A*. *catenella*, and three control groups were fed with the non-toxic microalgae *Alexandrium tamarense*. Each group was exposed the corresponding alga for 0, 4, 8, 16, 24, and 48 h (Fig 1). At the corresponding sampling times, approximately 1 ml of hemolymph was collected from the adductor muscle using a sterile 1 mL syringe, with a different syringe used for each mussel. Each hemolymph sample was centrifuged at 1200 x g for 20 min at 4°C to separate the hemocytes from the aqueous material. Then, supernatants were discarded, and 1 mL of the TRIzol reagent (Invitrogen, Life Technologies, USA) was added. Samples were then stored at -80°C until the entire experimental period ended. Stored samples were lysed in a Retsch MM 200 Mixer Mill (Retsch Inc., Germany) at 20 Hz for 5 min. Then, the standard protocol for RNA extraction with the TRIzol reagent was carried out following the manufacturer’s instructions. The phases were separated with 100% chloroform, and nucleic acid was precipitated with 100% isopropanol after cooling at -20°C. The extracted total RNA was washed three times with 75% ethanol cooled to -20°C and then treated with DNAse I (Thermo Scientific, USA) to eliminate genomic DNA contamination. RNA concentration and purity were measured in an ND-1000 spectrophotometer (Nanodrop Technologies, Thermo Scientific), and integrity was visualized by electrophoresis on a 1.2% MOPS-agarose gel under denaturing conditions. The purified RNA was stored at -80°C until later use. The first cDNA strain was retro-transcribed from 200 ng of purified RNA using the RevertAid H Minus First Strand cDNA Synthesis kit (Thermo Fisher Scientific, USA) according to the manufacturer’s instructions. This process included the addition of ribonuclease inhibitors to prevent the degradation of RNA used in synthesis.

### Selection and RT-qPCR expression analyses of candidate genes

From the transcriptomic analyses described above, seven candidate genes were selected for gene transcription qPCR runs (Table 1). Primers were designed with the commercial Geneious v8.0 software (Biomatters Ltd., New Zealand). PCR analyses were carried out using 1.5 U of the Taq polymerase enzyme (Thermo Fisher Scientific, USA) in a final reaction volume of 25 μL that included 1X enzyme buffer, 0.2 μg*μL^-1^ BSA, 1.5 mM MgCl_2_, and 500 nM of the respective primers. PCR cycles were run as follows: initial denaturing at 95°C for 5 min, followed by 35 denaturing cycles at 95°C for 30 s, annealing at 60°C for 30 s, extension at 72°C for 30 s, and a final extension at 72°C for 7 min. Amplification products were visualized on 1.2% agarose gels stained with 0.001% GelRed reagent (Biotium Inc., USA).

**Table 1:** List of candidate genes used in this study, including details on molecular function, primers, and reference.

Gene	Molecular function	Primers (5’-3’)	Reference
*PGRP*	Pattern Recognition Receptor	CACGAGGAGAATGTGTCAGG	Designed in this study
		ACGAAACTGTAGCCGATGTC	
*Lectin*	Pattern Recognition Receptor	CCGAACATGACGGTCTTGAT	Designed in this study
		TGGGAAAATTGTCCATCCCC	
*TLR-α*	Pattern Recognition Receptor	ACAAGCAACCAGGCTAAACA	Designed in this study
		ACTTTTGGACCATGCCAACT	
*Ikβα*	NF-κB pathway	GCATGCAATATGAAGTGGCG	Designed in this study
		GCATGCAATATGAAGTGGCG	
*IFN*	Interferon	GCCGGTTAGCTAGGAAGAAC	Designed in this study
		ACCAAAGGCTGTTCTCTTGG	
*TAK*	Signal transduction (TNF pathway)	TTCAGTTCAGTGCCTGTGAC	Designed in this study
		TCTCCTGTCTGTTGACTCGT	
*TRAF 6*	Signal transduction (TNF pathway)	GTGACCCAGTTCAAACACCT	Designed in this study
		ACGTTGTTCACCAGACTCAC	
*α-Tubulin*	Microtubule formation	GAGCCGTCTGCATGTTGAGC	Nuñez-Acuña *et al*., 2012
		TGGACGAAAGCACGTTTGGC	

Dynamic range analyses were conducted to obtain RT-qPCR efficiencies. The calibration curve for these runs consisted in five serial dilutions to obtain 200 ng of cDNA stock with a serial factor of 1:5. Each target gene was amplified with primers. The runs were performed with the Maxima SYBR-Green/Rox qPCR Master Mix 2X kit (Thermo Fisher Scientific, USA) according to the manufacturer’s instructions in a StepOne Plus Mastercycler (Applied Biosystems, Life Technologies, USA). For all runs, both primers were used at a concentration of 500 nM. The initial quantity of cDNA (80 ng) used for each reaction was based on dynamic range results. The same PCR run cycle as mentioned above was used, except that the annealing and extension steps were combined into a single step at 60°C for 60 s, followed by a holding step at 95°C for 10 min to activate the enzyme. After PCR analyses, a melting curve was constructed to verify the presence of a single amplification product and the absence of contaminants in the negative controls. The *a-tubulin* gene was used as an endogenous control based on prior validation as a housekeeping gene [18].

### Statistical analysis

Data were first assessed in Excel v2016 (Microsoft, USA); specifically, experimental and control single and biological group data were reordered, and the RQ value was manually calculated using the ΔΔCt formula [26]. Subsequently, the data were exported to the Statistica v.8.0 software (Statsoft, USA). To determine data distribution, a Shapiro-Wilk’s test was conducted for every analyzed gene. Then, the distribution and significant differences of data were evaluated by one-way ANOVA tests for parametrically distributed data, while the Kruskal-Wallis test was used for data with non-parametric distribution. In both cases, significant differences between biological groups were established at a value of p < 0.05.

## Results

### High throughput sequencing and transcriptome annotation of *Mytilus chilensis* injected with STX

**Fig 2:**
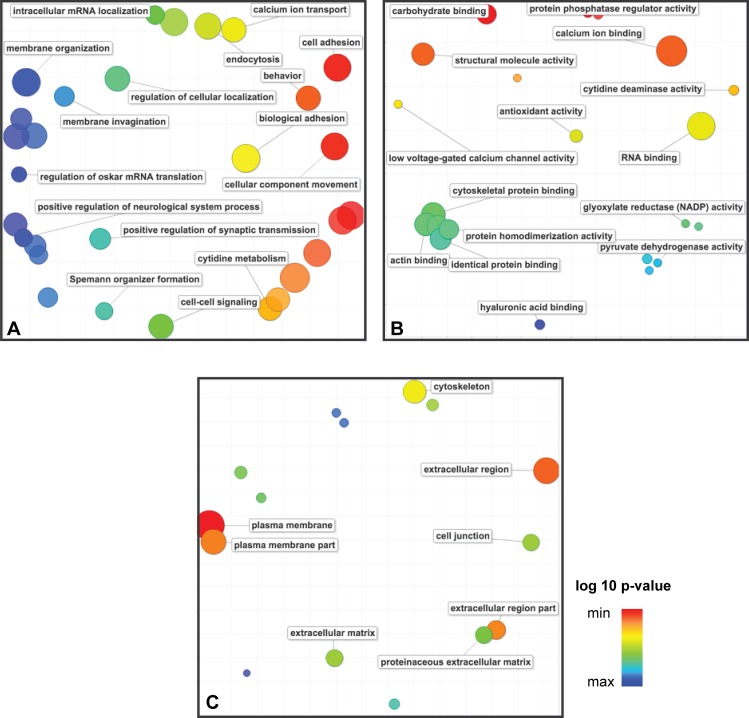
Gene Ontology (GO) enrichment analysis summarized as a scatter plot using REVIGO. (A) Summarized GO terms related to biological processes. (B) Summarized GO terms related to molecular function. (C) Summarized GO terms related to cellular components. GO terms are represented by circles and are plotted according to semantic similarities to other GO terms (i.e. adjoining circles are most closely related). Circle size is proportional to the frequency of the GO term, whereas color indicates the log10 P-value (red higher, blue lower).

Hemocytes of *M*. *chilensis* injected with STX were prepared and sequenced using the HiSeq Illumina platform. After quality trimming with the CLC Genomic Workbench v8.0 software, the sequencing run yielded a total of 799,899,594 reads with an average length of 101 bp. Of these, 591,732,138 reads (74%) were assembled into contigs, while 208,167,456 reads remained singletons and were excluded from further analyses. *De novo* assembly yielded 138,883 contigs with an average length of 504 bp. (Table 2). Then a BLASTx search against the UniProt Mollusca database was performed, demonstrating that ~30% of the contigs included in the non-redundant reference transcriptome were similar to Mollusca protein sequences. The sequenced genes of the annotated transcriptome were subjected to Gene Ontology analysis to determine corresponding functional categories. The 41,258 identified transcripts were classified by biological process, molecular function, and cellular localization (Fig 2). Gene Ontology biological processes related to cellular adhesion and cellular component movement were overrepresented. Interestingly, the most represented molecular processes and cellular components were calcium ion binding and the plasma membrane, respectively, suggesting an enrichment of membrane proteins after STX inoculation in mussels.

**Table 2:** Report of *Mytilus chilensis* transcriptomic sequencing.

Item	Obtained results
Number of reads	799,899,594
Average read length	101 bp
Number of contigs	138,883
Average contig length	504 bp
Number of assembled reads	591,732,138
Number of unmapped reads (singletons)	208,167,456

High-throughput data (trimmed reads) is available at Genbank database under accession number SRR3591491 of the SRA archive.

### RNA-seq expression analyses

#### Expression level of the whole transcriptome

**Fig 3:**
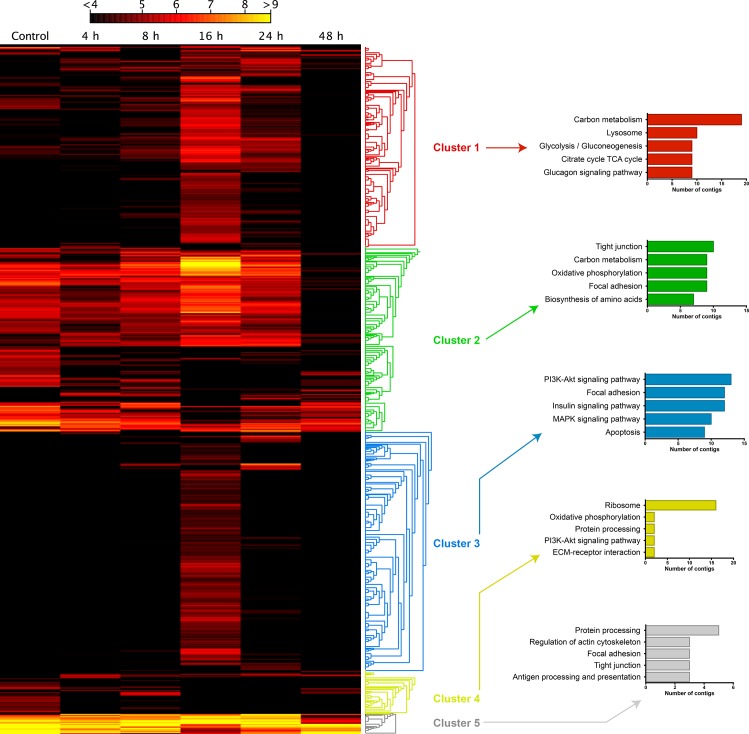
*In silico* gene transcription analysis at different times post-saxitoxin injection. Hierarchical clustering of transcripts according to obtained TPM values. Clusters were generated according to the similarity of expression patterns of different transcripts evaluated by Manhattan distances and the complete linkage test. For each cluster, the five most represented pathways are shown (KEGG pathway).

In addition to obtaining gene annotations for *M*. *chilensis*, a major aim of this transcriptomic study was to analyze the overall gene expression profile to identify transcripts involved in the molecular response of *M*. *chilensis* after exposure to STX. Following *de novo* assembly, contigs that showed matching reads for all samples, and with a fold change greater than |4| and a p < 0.01 at least in one condition, were used to create a new reference dataset (S1 Table). Then, gene expression data were normalized for six RNA-seq experiments to individually compare expression levels between the control group and the different experimental STX groups (i.e. 4, 8, 16, 24, and 48 hpi). A clustering analysis was conducted for 1,281 significantly expressed transcripts and showed differentiated transcription values between the different sampling time points (Fig 3).

**Fig 4:**
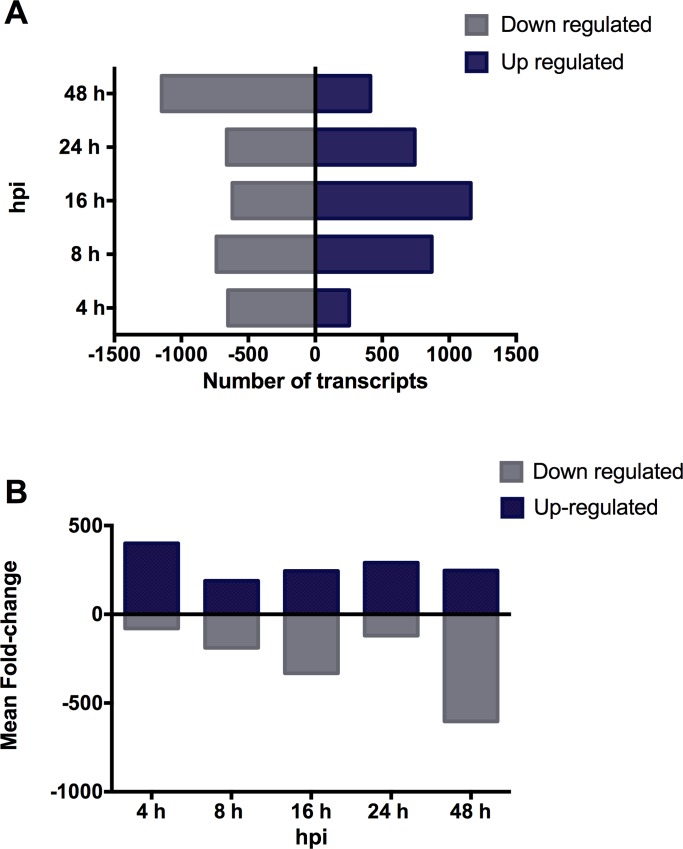
Transcriptome response after inoculation with STX. (A) Number of transcripts significantly up or down regulated according to p-value < 0.01 and fold change > |4| criteria from *Mytilus chilensis* at 4, 8, 16, 24 and 48 hours post inoculation with STX. (B) Fold- changes mean values from up or down regulated transcripts identified at each time post inoculation.

Through this analysis, five different clusters were identified. Transcripts composing Cluster 1 (323 contigs) were especially up regulated 16 and 24 hpi, but down regulated at 48 hpi. According to the KAAS tool, Cluster 1 transcripts were mainly involved in metabolic pathways, such as in carbon metabolism, lysosomes, glycolysis, the tricarboxylic acid cycle, and the glucagon signaling pathway. Cluster 2 (360 contigs) contigs were found involved in metabolism pathways as well as in cellular processes such as focal adhesion, tight junction, and amino acid biosynthesis. While the pattern was less clear than in the first cluster, Cluster 2 also evidenced a strong expression of most genes before 48 hpi. Cluster 3 (477 contigs) showed a strong, clear pattern of up regulated transcripts involved in apoptosis and signaling pathways at 16 hpi. In turn, Cluster 4 (73 contigs) transcripts involved in the ribosome pathway were down regulated after STX injection, whereas Cluster 5 (48 contigs) transcripts involved in cellular processes, such as cytoskeleton regulation or cell-cell communication, maintained high expressions even after injection. Interestingly, Clusters 1 and 3 evidenced particularly up regulated differential transcription activities at 16 hpi. In general, more transcripts were up regulated at 16 hpi than at other sampling time points, whereas the amount of down regulated transcripts was highest at 48 hpi (Fig 4).

#### Expression levels of immune receptors and ion channels

**Fig 5:**
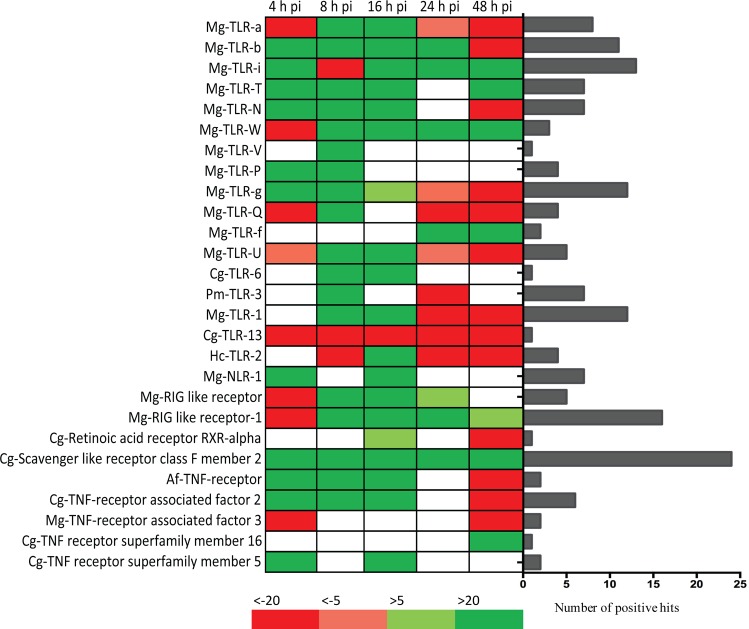
Selection of most significant PRR. (A) Most up- and downregulated pattern recognition receptors (PRRs) at the five post-injection sampling times (4, 8, 16, 24, and 48 h) compared to the control and according to corresponding fold changes. Green indicates a fold change (FC) greater than 20; light green, FC > 5; red, FC < -20, and in light red, FC < -5. (B) Number of positive hits for each PRR transcript.

**Fig 6:**
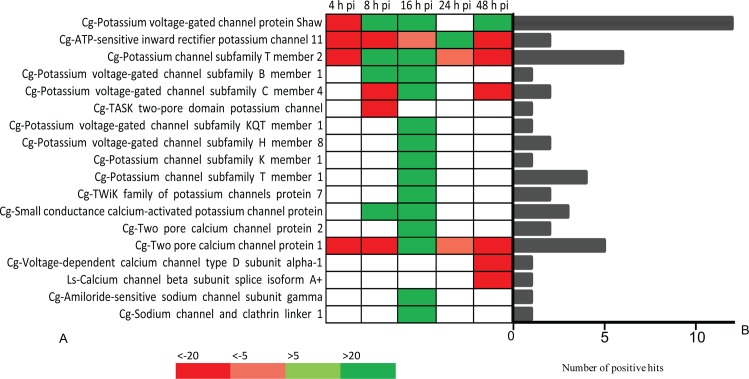
Selection of most significant ions channels. (A) Most up and downregulated channels at the five post-injection sampling times (4, 8, 16, 24, and 48 h) compared to the control. Green indicates a fold change (FC) greater than 20; light green, FC > 5; red, FC < -20, and in light red, FC < -5. (B) Number of positive hits for each channels transcripts.

To understand how mussels can recognize and defend against STX, the impacts of this toxin on *M*. *chilensis* channels and PRRs were assessed. Significant (FC > |4| and p-value < 0.01) receptor and channel transcripts were selected to create a new reference dataset, and the expression level of each transcript was compared at six different sampling time points (S1 Fig), resulting in the identification of 1,108 significantly expressed transcripts belonging to immune receptors or channels. From these, the most up- and down regulated PRRs were selected according to fold change (FC > 20 or FC < -20, for at least one sampling time point) (Fig 5). Finally, 27 PRRs were found highly up- or down regulated for at least one sampling time. Of these, 63% represented TLRs and 19% tumor necrosis factor (TNF) receptors, while the remaining PRRs were nucleotide-binding oligomerization domain-like (NOD-like) receptors, scavenger receptors, retinoic acid-inducible gene-like (RIG-like) receptors, and retinoic acid receptors. The number of positive hits for these receptors varied from two for TLR 5, 6, and 13; for the retinoic receptor; and for the TNF receptor superfamily member 16, to 24 for the scavenger-like receptor class F member 2. Interestingly, 67 and 70% of PRRs were found upregulated at 8 and 16 hpi, respectively. The same analysis was performed on ion channels (Fig 6), with 18 transcripts found highly regulated post-injection. Of these, 67% were potassium channels, while 22 and 11% were calcium and sodium channels, respectively. Worth noting, most sodium channels were down regulated at 4 and 48 hpi, while, in contrast, 78% of the most significant channels were up regulated at 16 hpi. To ensure that the response of PRRs and channels was not due to the wounding effect of the injection, the expression of five immune genes (TLR-i, Galectin, TNF receptor, Mytilin B, Ependymin) and one potassium channel gene was investigating in mussels injected with STX and mussels injected with PBS (S2 Fig). Interestingly, the up regulation of immune genes occurs only in mussels injected with STX, suggesting a specific response to the toxin.

#### Pathways

**Fig 7:**
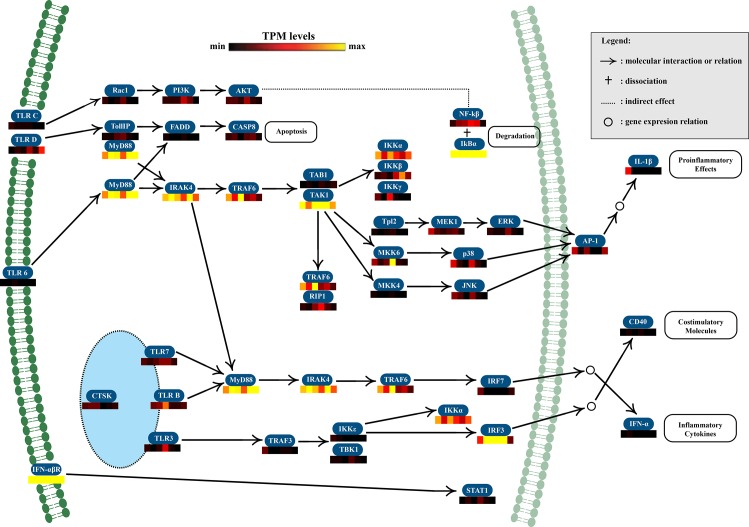
Putative pathway of *Mytilus chilensis* Toll-like receptor (TLR) gene cascade. The scheme shows the most probable interactions between different transcripts according to KEGG pathway criteria. Additionally, transcription levels of each gene are shown according to TPM values to evaluate corresponding expression patterns in response to saxitoxin injection. Transcriptions patterns were evaluated 0, 4, 8, 16, 24, and 48 h post-injection. Light-blue ellipse represents the endosome.

**Fig 8:**
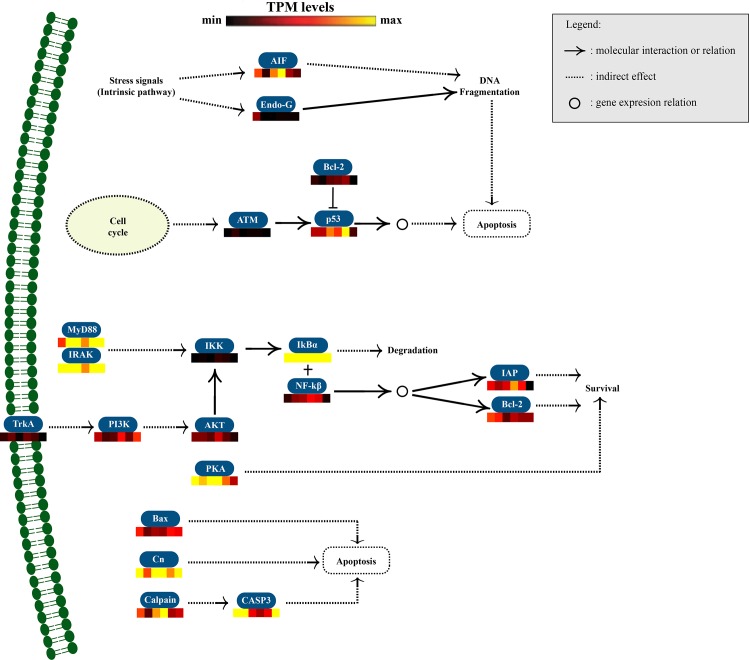
Putative pathway of *Mytilus chilensis* apoptosis gene cascade. The scheme shows the most probable interactions between different transcripts according to KEGG pathway criteria. Additionally, transcription levels of each gene are shown according to TPM value to evaluate corresponding expression patterns in response to saxitoxin injection. Transcription patterns were evaluated 0, 4, 8, 16, 24, and 48 h post-injection.

Previous PRR analyses revealed strong TLR and TNF responses following STX injection. TLRs are involved in the primary immune pathway of invertebrates (i.e. the Toll pathway), while TNF receptors are mainly involved in apoptosis. Therefore, other transcripts involved in these two pathways were analyzed (Figs 7 and 8). TLR B up regulated from 4 to 24 hpi, was linked to MyD88, IRAK4, and TRAF6, which were strongly expressed at different post-injection time points according to TPM values. Likewise, TLR-6, which was up regulated at 8 and 16 hpi, seemed to activate a cascade of proteins, including MyD88, IRAK4, TRAF6, and TAK1. Additionally, TRAF6 and TAK1 were also strongly expressed at different post-injection sampling times.

Genes involved in the apoptosis pathway also exhibited differential expression levels (Fig 8). The apoptosis inducing factor, one of the most important apoptosis pathway genes, was strongly up regulated, especially at 16 hpi. This gene leads to DNA fragmentation and, indirectly, to cellular apoptosis. The p53 gene was also upregulated at 24 hpi. Both the apoptosis inducing factor and p53 genes are involved in the intrinsic pathway that can drive a cell into apoptotic stages. Furthermore, other genes related to an independent apoptotic signaling pathway were upregulated, such as Bax, Cn, Calpain, and Caspase-3.

### qPCR analyses of specific gene expressions

**Fig 9:**
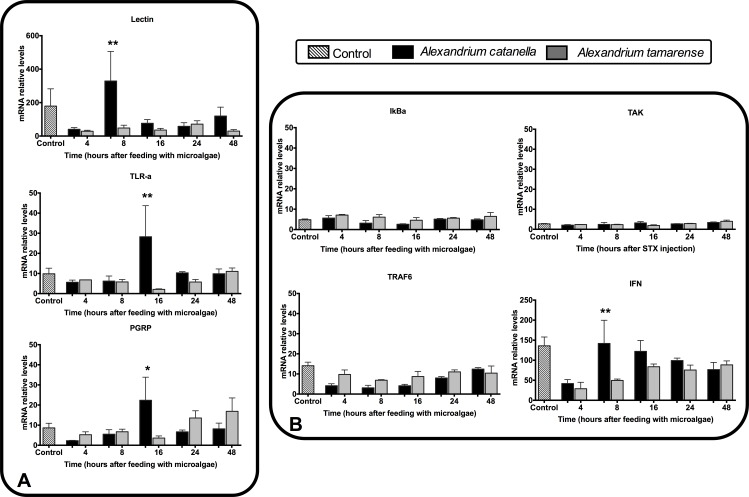
Gene transcription analysis of selected PRRs transcripts. (A) Transcripts involved in immune pathways, and (B) in response to the presence of *Alexandrium caetenella*. *Alexandrium tamarense* was used as a control. The transcripts were assayed through qPCR at different exposure times to the microalgae (0, 4, 8, 16, 24, and 48 h). * significantly different from the non-toxic algae at the same time point (* p-value < 0.05, ** p-value < 0.01, ANOVA).

To assess the transcriptional responses of immune genes to the toxic microalgae *A*. *catenella*, qPCR analyses were performed on seven immune genes, with the nontoxic microalgae *A*. *tamarense* used as an experimental control. The main goal of this specific analysis was to evaluate the immune response of the mussel to the whole microalgae, not only to the toxin. Studied genes were categorized into two groups, with the first comprised of diverse PRRs (i.e. TLR-a, PGRP, and lectin) and the second comprised of signaling genes involved in immunity (i.e. IkBa, IFN, TAK, and TRAF6) (Fig 9). The primary lectin response occurred at 8 h post-challenge, evidencing significant up regulation while PGRP and TLR-a significantly responded 16 h post-challenge (Fig 9A). In contrast to TLR-a, the IkBa, IFN, TAK, and TRAF6 genes did not respond to the toxic algae (Fig 9B). Similarly, the nuclear protein IkBa seemed unregulated after the *A*. *catenella* challenge as compared to nontoxic microalgae treatment. However, IFN have a significant increase of expression at 8 h post-challenge.

## Discussion

Numerous studies have focused on the physiological and cellular reactions of bivalves to PST exposure. In bivalves, toxins may reduce growth and reproduction, decrease filtration rates, and induce changes in respiration [10–12]. As sentinel species, bivalves are of particular investigative interest not only due to PST sensitivity, but also since these organisms are capable of accumulating high toxin concentrations in the digestive gland [27, 28]. However, the effects of toxic algae on bivalve immune systems are still not completely understood. Bivalve immune responses can be affected by harmful microalgae or the toxin inherent to these species [29–41]. Recently, the responses of *M*. *chilensis* immune candidate genes were examined following *in vivo* [18] and *in vitro* [17] STX exposure.

Similarly, the present study examined *in vivo* the effects of purified STX (i) on the whole *M*. *chilensis* transcriptome, and (ii) on specific membrane-bound genes, such as PRRs and ion channels, the results of which were complemented by (iii) investigating the effects of *A*. *catenella* on the specific immune transcription of hemocytes in *M*. *chilensis*. Whole transcriptome analysis revealed five clusters categorized by contigs expressions after STX inoculation. Cluster 3 was of particular interest as most of the cluster contigs were linked to signaling pathways or apoptosis and were upregulated at 16 hpi. Moreover, the significant enrichment of calcium ion binding proteins and membrane proteins revealed a potential effect of STX on calcium channels and membrane receptors. These results concur with prior results showing a negative impact of STX on calcium binding proteins, such as the voltage-dependent calcium channel, in mammals and marine species [7, 42]. However, the results of the current study evidenced a potentially more complicated response of the most regulated channels.

Although the voltage-dependent calcium channel type D was down regulated at 48 hpi, the response of other calcium channel proteins varied. The two-pore calcium channel protein 1 was significantly down regulated at 4, 8, 24, and 48 hpi and significantly up regulated at 16 hpi. In turn, the two-pore calcium channel protein 2 was only up regulated at 16 hpi. Moreover, most of the potassium channels were up regulated at 16 hpi and down regulated at the beginning (4 hpi) and end (48 hpi) of the experimental period. These results suggest early and late negative potassium and calcium channel-related responses and positive responses at 16 hpi. We hypothesize that the up regulation of most channels at 16 hpi is an attempt of the organism to maintain cellular ion homeostasis.

Membrane protein enrichment could be related to the significant up regulation of some PRRs, primarily at 8 and 16 hpi. PRRs are families of extracellular, membrane-bound or cytosolic immune receptors capable of inducing a finely-tuned expression of genes referred to as immune effectors (e.g. antimicrobial peptides, cytokines, chemokine) or of triggering a cell response to infection (e.g. phagocytosis, apoptosis) in mussels [43]. As a response to STX exposure, the present study identified the expression of various PRRs, including 17 TLRs and some TLR-related signaling proteins, such as IRAK, TAK1, and TRAF6. The TLR pathway is well-described in model invertebrates (e.g *Drosophila*) [44], and, more recently, in mussels [45] as one of the main immune pathways responding to bacterial and fungal infections. Nevertheless, in most marine invertebrates, the functional role of each TLRs, is still unclear. Here, we suggest that the TLR pathway could also be involved in recognizing and defending organisms against toxins.

Other membrane PRRs were also found expressed in response to STX exposure, including scavenger-like receptors that were consistently up regulated from 4 to 48 hpi. Scavenger-like receptors have been identified in several bivalves, but have only been fully characterized in one bivalve, showing an ability to bind various ligands (e.g. lipopolysaccharides, peptidoglycans, mannans, and zymosans) [46]. Differentially expressed cytosolic PRRs included retinoic acid-inducible gene-like receptors and NOD-like receptors, which play major roles in recognizing viruses and intracellular bacteria, respectively [24]. In this study, retinoic acid-inducible gene-like receptors positively responded to STX at 4 hpi, and one NOD-like receptor was up regulated at 4 and 16 hpi. NOD-like receptors are crucial for the activation of autophagic machinery [43]; therefore, the up regulation of a NOD-like receptor post-injection suggests an induction of immune cellular processes, particularly autophagy, to limit the toxic effects of STX. Likewise, TNF receptors, cytokines involved in immunity and the extrinsic apoptosis pathway [47], were upregulated at 4, 8, 16, and 24 hpi, possibly acting as a secondary cellular mechanism to eliminate toxin-damaged cells. Together these results suggest, for the first time, that PRRs may be able to specifically recognize abnormal, foreign elements, such as toxins, and trigger an immune response that allows *M*. *chilensis* to prevent or cope with detrimental STX effects.

A second focus of this study was to determine the effects of *A*. *catenella* on specific immune transcripts of hemocytes in *M*. *chilensis*. Two gene categories were investigated, a group of PRRs and a group of signaling proteins involved in immune pathways. The first PRR that responded to toxin exposure was lectin, followed at 16 h post-exposure by TLR-a and PGRP. Lectins are extracellular receptors involved in the encapsulation of bacteria [48], while, in bivalves, TLR-a and PGRP are membrane receptors involved in the Toll and IMD pathways, respectively [45, 49]. The expression of TLRs and C-type lectin in hemocytes after exposure to *A*. *catenella* has been previously investigated *in vitro* [17]. Interestingly, *in vivo* the response of hemocytes occurs earlier, with an acute response at 8 and 16 h post-exposure. This is in contrast to *in vitro* experiments showing a down regulation of these PRRs before 16 h and an up regulation between 24 and 48 h post-exposure [17].

In the second group of assessed genes, IFN was the only signaling transcript significantly regulated after exposure to toxic algae. IFN is a cytokine-like molecule that plays significant roles in mediating the virus immune response [50]. In turn, TAK1, TRAF6, and IKba are linked to the Toll and NF-KappaB pathways. It is possible that both anti-pathogen (bacteria and fungi) and anti-virus receptors recognize the toxic algae, but that only anti-virus effectors are produced due to superior defense mechanisms against toxic algae. Overall, the obtained qPCR expression patterns of the selected genes in mussels exposed to *A*. *catenella* suggest as for mussels injected with STX, the involvement of some PRRs in the recognition mechanism.

## Conclusions

The present study theorized that the response of *M*. *chilensis* to an *in vivo* injection of purified STX involves numerous PRRs. The expression of these PRRs seems specifically correlated to the toxin and subsequently could trigger either (i) a signaling response via the Toll pathway, or (ii) a cellular response via autophagy or apoptosis. The identified PRRs can recognize PAMPs from bacteria, fungi, or viruses. This study demonstrated that PRRs such as TLR, retinoic acid-inducible gene-like receptors, NOD-like receptors, TNF-receptors, and scavenger-like receptors may be able to recognize STX as an invasive, foreign molecule, which could act as a PAMP-type molecule. These receptors are responding to a relatively low concentration of STX (80 μg of STX / 100 g of wet meat) while at the present time in Chiloé island the concentration of STX in shellfish reached 8000 μg / 100g of wet flesh. This bloom of toxicity has led to massive deaths and hence major environmental, economical and social issues in Chile. Here, we supposed that the immune response could be dose dependent, and that greater concentrations than the one used could make the system collapse leading to mussels’ death.

Interestingly, the response *M*. *chilensis* to toxic algae-exposure appeared to be triggered by other PRRs, such as lectin and PGRP, suggesting recognition of molecules from the algal cell wall. Due to the lack of continuous control in the STX injection experiment, further analysis are needed to validate and deeply investigate the molecular interactions of these receptors with STX. Nonetheless, the presently provided insights into the molecular mechanisms that mussels deploy to defend against STX and toxic microalgae represent novel contributions towards understanding the biological implications of harmful algal blooms in marine environments.

## Supporting Information

S1 Fig *In silico* gene transcription analysis of pattern recognition receptors (PRRs) and channels at different times post-saxitoxin injection. Hierarchical clustering of significant receptors and channels transcripts is shown (fold change > 4 and p-value < 0.01) according to obtained TPM values. Clusters were generated according to the similarity of the expression patterns of different transcripts evaluated by Manhattan distances and a complete linkage test.
(DOCX)

S2 Fig Gene transcription analysis of selected transcripts. Selected immune and channels transcripts involve in the response to the toxin. The transcripts were assayed through qPCR at different hours post inoculation (4, 8, 16 and 24). White bar correspond to mussels injected with saline solution (PBS) (control), while red bars shows the expression of mussels inoculated with STX.
(DOCX)

S1 Table Expression of all genes with a fold change > |4|. For each time point appear the Fold Change, the p-value and the False Discovery Rate (FDR).
(XLSX)
