# Supplementary material for: Uncovering the Complex Transcriptome Response of Mytilus chilensis against Saxitoxin: Implications of Harmful Algal Blooms on Mussel Populations
Source: PLoS One. 2016 Oct 20;11(10):e0165231. doi: 10.1371/journal.pone.0165231 (PMC5072731; doi:10.1371/journal.pone.0165231)
Supplement: Supplementary file 1 [file pone.0165231.s002.docx]

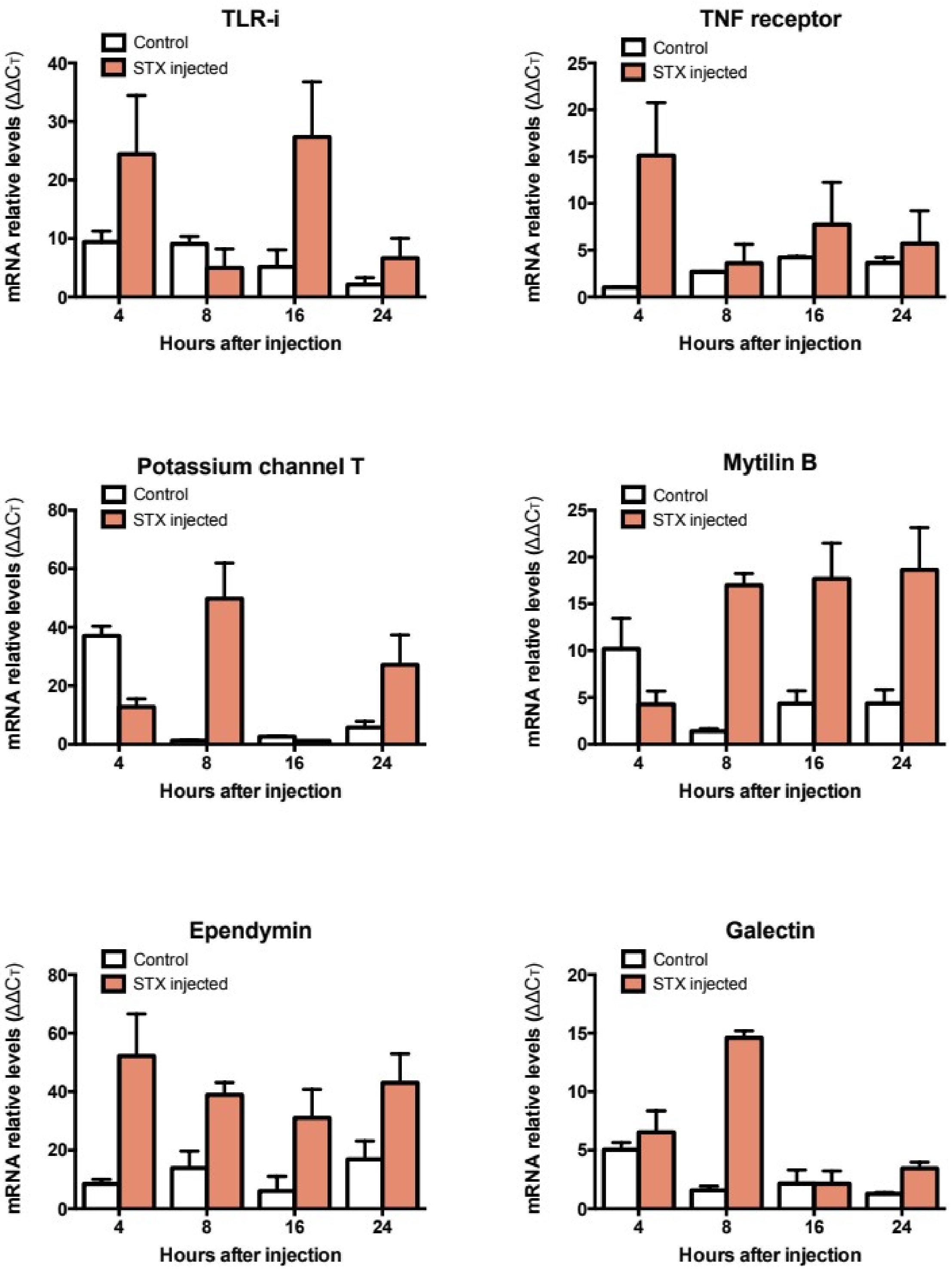
TLR-i
Control
STX injected
mRNA relative levels (ΔΔCT)
0
10
20
30
40
4
8
16
24
Hours after injection
TNF receptor
Control
STX injected
mRNA relative levels (ΔΔCT)
0
5
10
15
20
25
4
8
16
24
Hours after injection
Potassium channel T
Control
STX injected
mRNA relative levels (ΔΔCT)
0
20
40
60
80
4
8
16
24
Hours after injection
Mytilin B
Control
STX injected
mRNA relative levels (ΔΔCT)
0
5
10
15
20
25
4
8
16
24
Hours after injection
Ependymin
Control
STX injected
mRNA relative levels (ΔΔCT)
0
20
40
60
80
4
8
16
24
Hours after injection
Galectin
Control
STX injected
mRNA relative levels (ΔΔCT)
0
5
10
15
20
4
8
16
24
Hours after injection
